# Mechanisms linking obesity to altered metabolism in mice colon carcinogenesis

**DOI:** 10.18632/oncotarget.5561

**Published:** 2015-10-12

**Authors:** Lili Nimri, Janan Saadi, Irena Peri, Einav Yehuda-Shnaidman, Betty Schwartz

**Affiliations:** ^1^ Institute of Biochemistry, Food Science and Nutrition, The Robert H. Smith Faculty of Agriculture, Food and Environment, The Hebrew University of Jerusalem, Jerusalem, Israel

**Keywords:** obesity, colon cancer, leptin, mitochondria, JNK/STAT3

## Abstract

There are an increasing number of reports on obesity being a key risk factor for the development of colon cancer. Our goal in this study was to explore the metabolic networks and molecular signaling pathways linking obesity, adipose tissue and colon cancer. Using *in-vivo* experiments, we found that mice fed a high-fat diet (HFD) and injected with MC38 colon cancer cells develop significantly larger tumors than their counterparts fed a control diet. In *ex-vivo* experiments, MC38 and CT26 colon cancer cells exposed to conditioned media (CM) from the adipose tissue of HFD-fed mice demonstrated significantly lower oxygen consumption rate as well as lower maximal oxygen consumption rate after carbonyl cyanide-4-trifluoromethoxy-phenylhydrazone treatment. In addition, *in-vitro* assays showed downregulated expression of mitochondrial genes in colon cancer cells exposed to CM prepared from the visceral fat of HFD-fed mice or to leptin. Interestingly, leptin levels detected in the media of adipose tissue explants co-cultured with MC38 cancer cells were higher than in adipose tissue explants cultures, indicating cross talk between the adipose tissue and the cancer cells. Salient findings of the present study demonstrate that this crosstalk is mediated at least partially by the JNK/STAT3-signaling pathway.

## INTRODUCTION

Obesity caused by overnutrition and a sedentary life style leads to abnormal or excessive fat accumulation on the adipose tissue, induction of metabolic disorders and ultimately, impaired health [1]. Studies aimed at elucidating alterations in the mechanisms associated with obesity have found that mitochondrial dysfunction accompanies excess lipid accumulation and oxidative stress in several organs, such as the skeletal muscle and liver [2]. High insulin levels and insulin resistance have been shown to be directly associated with the suppressed mitochondrial function [3]. Mitochondrial suppression and dysfunction also characterize cancer cells as they use aerobic glycolysis and glycolytic intermediates to generate energy and enhance growth, a phenomenon called “the Warburg effect”. Cancer cells also generate important cofactors and redox components, such as high levels of reduced forms of NAD^+^, NADH, and NADPH. Altered metabolic activities like these can be linked to both mitochondrial oxidative phosphorylation (OXPHOS) and biogenesis inhibition, which can support a metastatic phenotype [4, 5].

It has been established that obesity can contribute to up to 20% of all cancers [6], including colon cancer, which is one of the prevalent forms of cancer worldwide [7]. Epidemiological studies have clearly shown a direct correlation between obesity and the relative risk of developing colon cancer [6]. *In-vivo* studies in mice have shown that a high-fat diet (HFD) increases the metastatic ability of colon cancer cells [8]. Adipocytes have been shown to promote tumor growth and invasion in breast and ovarian cancers in *in-vivo* and *in-vitro* models [9, 10]. Nevertheless, the specific molecular mediators responsible for the association between obesity and cancer are numerous and their putative effects are very complex, and therefore additional studies are needed to shed light on these important issues.

Recently, Tebbe *et al* [11] demonstrated that conditioned media (CM) prepared from adipocytes enhance the migration and proliferation of ovarian cancer cells. Our previous study [12] demonstrated that CM prepared from human visceral adipose tissue obtained from obese subjects induce a significant decrease in the mitochondrial function and respiration ability of human colon cancer cells. This effect was partly mediated by leptin, an adipocytokine secreted by the adipose tissue in correlation with fat mass [13]. Indeed, the association of leptin with cancer and obesity, including colon cancer, has been previously studied by us [12, 14] and others [15]. Leptin was pinpointed as a potential mediator between obesity and cancer. Leptin affects mitochondrial function and decreases the expression of mitochondrial genes [12].

Montague *et al* [16] previously demonstrated the marked overexpression of leptin mRNA transcripts in abdominal subcutaneous as compared to visceral adipocytes; however, the visceral adipose tissue (VAT) depot still contained a higher number of proinflammatory macrophages [17, 18]. These contrasting findings led us to investigate which fat depot is responsible for promoting cancer cell growth and progression.

Based on our previous findings [12], we hypothesize herein that obesity promotes colon cancer primarily by causing mitochondrial dysfunction and decreasing OXPHOS gene expression. In order to verify this hypothesis we used *in-vivo*, *in-vitro* and *ex-vivo* models and demonstrated that a HFD can promote cancer progression in mice, and concomitantly induce mitochondrial dysfunction in several relevant organs. We also show that products secreted from CM prepared from mouse VAT promote mitochondrial dysfunction of cancer cells, and that this effect is mediated by the c-Jun N-terminal kinase (JNK)/STAT-3-signaling pathway. We conclude that this pathway may play an important role in the relationship between obesity and colon cancer.

## RESULTS

### HFD induces tumor growth in mice injected with MC38 colon cancer cells

**Figure 1:**
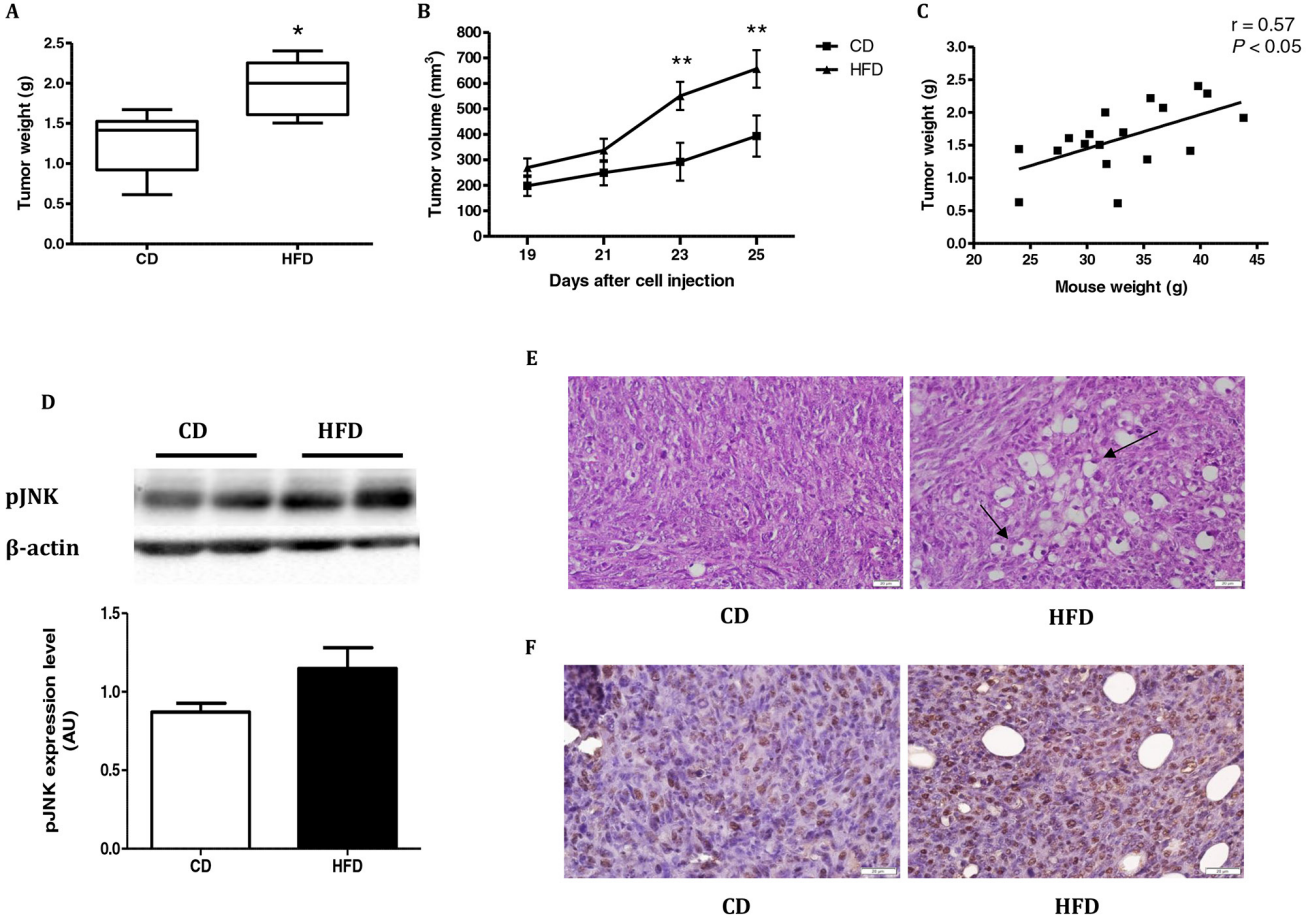
High-fat diet (HFD) stimulates tumor growth in C57BL/6 mice injected with MC38 cells **A.** Tumor weight and **B.** tumor volume were measured; *n* = 9 from the control diet (CD) group and *n* = 9 from the HFD group. *P < 0.01 (Student's unpaired *t-*test), **P < 0.05 (two-way ANOVA–Bonferroni post-test). **C.** Simple linear regression between tumor weight in the two groups and mouse weight was performed using Pearson's r test (*n* = 18, *r* = 0.57, *P* < 0.05). **D.** Tumor tissues from the two groups were analyzed by western blot and densitometry analysis of the western blot data was performed (*n* = 7 for CD, *n* = 7 for HFD). **E.** H&E staining of the tumors sections from each group. Arrows indicate large lipid droplets and high nuclear density. Scale bar = 20 μm. This is one representative section from one mouse out of four different mice. **F.** Immunohistochemistry was performed on tumors from each group using anti-PCNA antibody. PCNA protein is localized in the cell nucleus of the tumors. Nuclei were counterstained with hematoxylin. Scale bars = 20 μm. This is one representative section from one mouse out of four different mice.

The effects of HFD on mouse physiology were measured and are shown in Supplementary Fig. S1A–F and Supplementary Table S1. Mice fed HFD gained more weight than those fed a control diet (CD); moreover, HFD-fed mice were insulin-resistant, even though there was no difference in food intake between the two groups (Supplementary Fig. S1A–F). Leptin levels were significantly higher in the HFD-fed mice at the end of the experiment, as were weight and fat mass (Supplementary Table S1). Four weeks after MC38 cells injection, mice were sacrificed and the tumors were collected. Tumor weight (Fig. 1A) and tumor volume (Fig. 1B) were significantly higher in mice fed the HFD vs. CD. A positive linear regression (*P* < 0.05) was obtained between the weights of the mice from the two groups and their respective tumor weights (Fig. 1C). Western immunoblot analyses of tumor samples revealed higher pJNK levels in mice fed HFD as compared to tumor samples from mice fed CD (Fig. 1D). Hematoxylin and eosin (H&E) staining (Fig. 1E) and immunostaining with anti-proliferating cell nuclear antigen (PCNA) antibody [19] (Fig. 1F) revealed the presence of large lipid droplets, high nuclear density and strong PCNA staining in the tumor sections from the mouse group fed the HFD. These results demonstrated enhanced proliferation of cancer cells in the tumors of HFD-fed mice, and the concomitant accumulation of fat between the cells which might play a role in tumorigenesis.

### CM collected from adipose tissue of mice fed HFD inhibit respiration of MC38 and CT26 cells

Exploring the effect of adipose tissue on cancer progression, we have previously shown that secreted products from the adipose tissue of obese subjects inhibit mitochondrial respiration and function in HCT116 human colon cancer cells, and that the effect is at least partly mediated by leptin [12].

**Figure 2:**
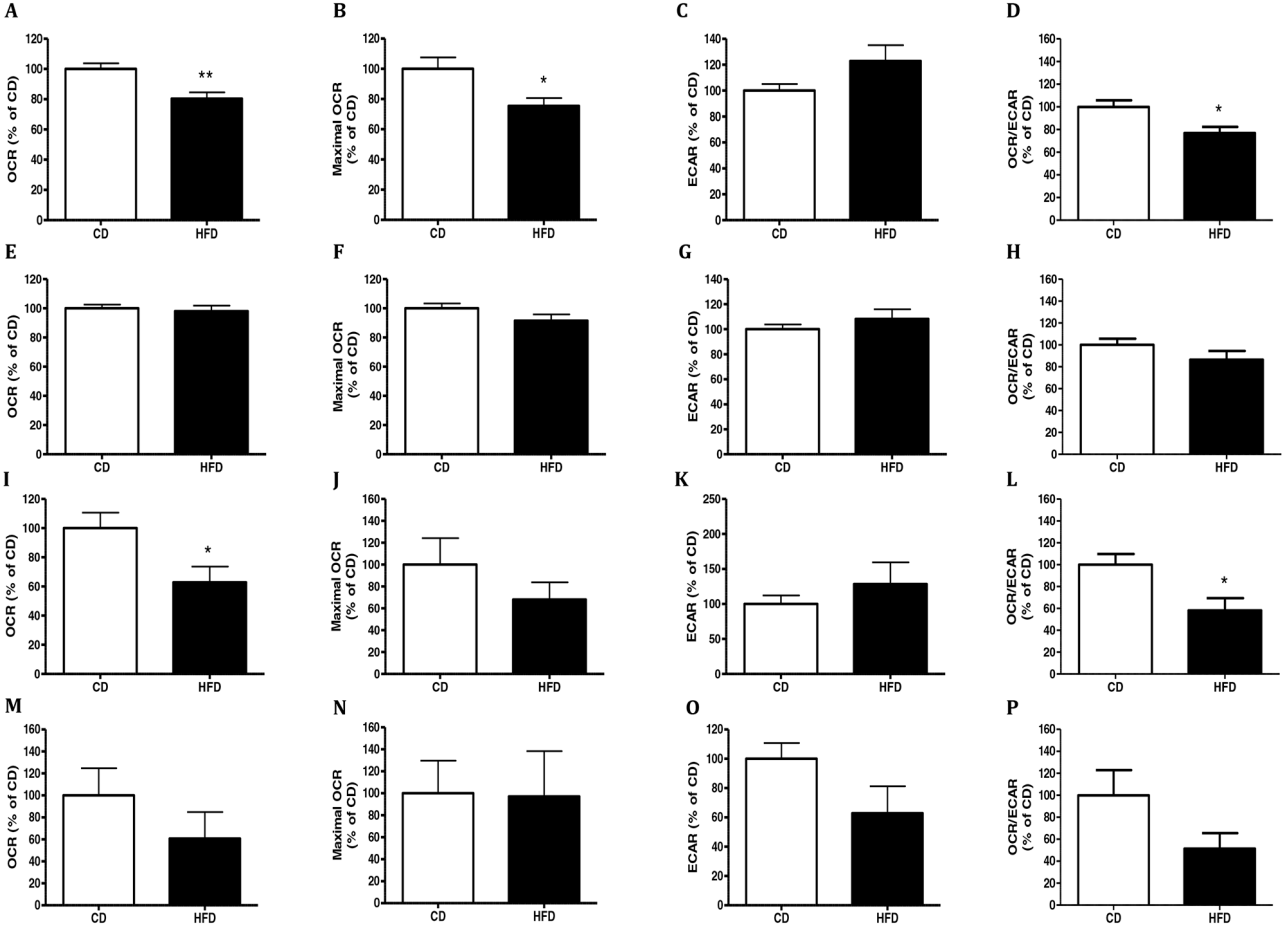
Conditioned media (CM) from HFD-fed mice decreased cells respiration MC38 **A–H.** and CT26 **I–P.** cells were treated for 24 hours with conditioned media (CM) collected from visceral adipose tissue (VAT) (A-D, I-L) or subcutaneous adipose tissue (SAT) (E-H, M-P) of mice fed control diet (CD; *n* = 4) or high-fat diet (HFD; *n* = 4) and analyzed for their oxygen consumption rate (OCR), extracellular acidification rate (ECAR), OCR/ECAR ratio and maximal OCR following administration of 0.4 μM FCCP using the XF24 Analyzer. *P < 0.05, **P < 0.01 vs. CD mice (Student's unpaired *t*-test). Results of two independent experiments are shown.

In order to prepare CM we used fat depots from mice fed HFD or CD for 16 weeks (Experiment 2 in materials and methods). To this end we removed visceral adipose tissue (VAT) or subcutaneous adipose tissue (SAT) from mice and incubated MC38 or CT26 murine colon cancer cells with the different CM. We tested the effects of CM on the glycolytic activity and mitochondrial respiration of the cancer cells. We used MC38 and CT26 murine colon cancer cells since they are two distinctly aggressive, syngeneic cancer cell lines. They both express the MYB oncoprotein, a protein usually over expressed in colon cancer and essential for continued proliferation and tumor cell survival [20]. As shown in Fig. 2, MC38 or CT26 cells exposed to CM of VAT from HFD-fed mice exhibited significantly lower oxygen consumption rate (OCR) (*P* < 0.01 and *P* < 0.05, respectively) than cells exposed to CM prepared from mice fed CD (Fig. 2A and 2I, respectively). In addition, the maximal OCR measured using the mitochondrial uncoupler carbonyl cyanide-4-trifluoromethoxy-phenylhydrazone (FCCP) was also significantly reduced in MC38 cells exposed to CM prepared from mice fed HFD, and the same trend was shown in CT26 cells but with no significance (Fig. 2B and 2J, respectively). In contrast, OCR inhibition was not observed when the cancer cells were incubated with CM of SAT prepared from mice fed HFD (Fig. 2E, 2F, 2M and 2N). No effects of CM were detected on were detected on extracellular acidification rate (ECAR) of MC38 or CT26 cells (Fig. 2C, 2G, 2K and 2O).

The OCR/ECAR ratio support the above mentioned results and show a significant (*P* < 0.05) reduction in OCR/ECAR after adding CM prepared from VAT of HFD- fed mice on MC38 and CT26 cells (Fig. 2D, and 2L, respectively). However, no significant change in OCR/ECAR ratio was observed when adding CM prepared from SAT of HFD–fed mice on the cells (Fig. 2H,2P).

To rule out lipotoxicity, we tested the effect of the different CM on the viability of MC38 cells. No differences were detected (Supplementary Fig. S2A and B).

These results suggested a strong inhibitory effect of visceral fat on the mitochondrial respiration of cancer cells, which may induce cancer-progression-associated mechanisms [21, 22].

### CM prepared from VAT of mice fed HFD decreases mitochondrial gene expression in CT26 colon cancer cells

**Figure 3:**
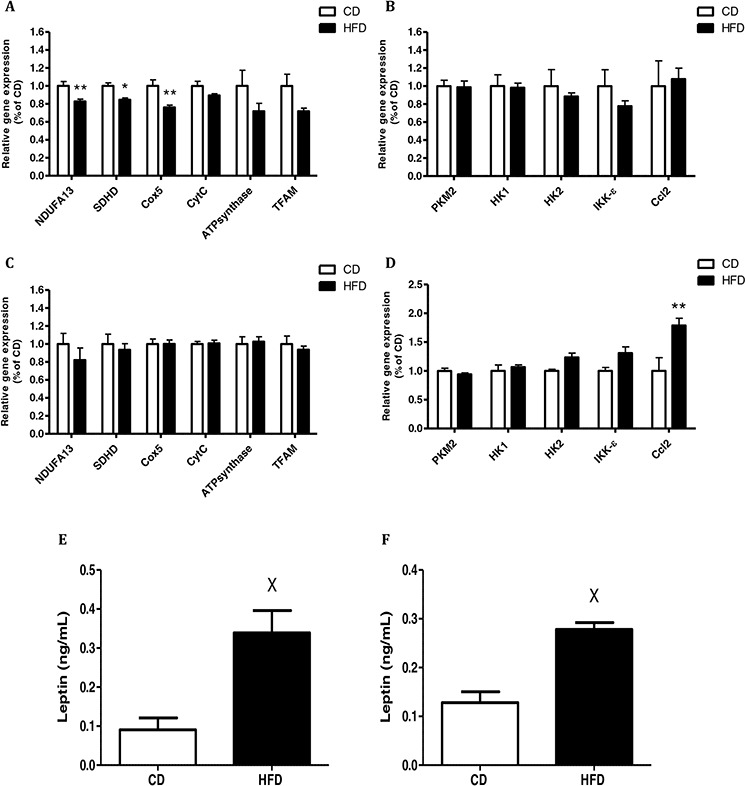
Conditioned media (CM) from high-fat diet (HFD)-fed mice decreased mitochondrial function and had a higher level of leptin CT26 **A, B.** and MC38 **C, D.** cells were treated with CM obtained from mouse visceral adipose tissue (VAT) for 24 hours (as described in Materials and Methods). Gene expression was detected using quantitative real-time PCR. *P < 0.01, **P < 0.05 vs. CD mice, *n* = 4 (Student's unpaired *t*-test). E, F, Leptin levels in CM prepared from VAT **E.** or subcutaneous adipose tissue (SAT) **F.** Leptin levels were measured using an ELISA kit; n = 4–5 mice from each group, ^X^*P* < 0.01 vs. CD (Student's unpaired *t*-test).

Continuing our exploration of the effect of CM from mice fed HFD on the mitochondrial and glycolytic activities of cancer cells, we examined the effect of CM from VAT on mitochondrial and glycolytic gene expression in CT26 cells, since these CM had a significant effect on these cells' respiration (see Fig. 2). CT26 cells exposed to CM prepared from VAT of mice fed HFD showed significantly diminished expression of the following mitochondrial genes: succinate dehydrogenase complex subunit D (*SDHD*), NADH dehydrogenase (ubiquinone) 1 alpha subcomplex gene (*NDUFA13*) and cytochrome c oxidase subunit 5B (*Cox5*) (Fig. 3A). In contrast, no change was detected in the expression of the glycolytic genes hexokinase1 (*HK1*), hexokinase 2 (*HK2*) or the tumor-specific isoform of pyruvate kinase M2 (*PKM2*) (Fig. 3B).

Interestingly, no effect on mitochondrial gene expression was seen with MC38 cells (Fig. 3C). However, a significant increase in expression of the chemokine (C–C motif) ligand 2 gene (*Ccl2*), which is also referred to as the gene encoding monocyte chemotactic protein 1 (*MCP1*), was measured in MC38 exposed to CM prepared from VAT of mice fed HFD. The same trend was obtained for I-kappa-B kinase epsilon (*IKK-* e) expression levels, but did not reach statistical significance (*P* = 0.07) (Fig. 3D). Collectively, these results suggested that CM prepared from VAT of obese mice can inhibit mitochondrial respiration in CT26 colon cancer cells, a change that is characteristic of the metabolic reprogramming typical of malignant transformation. In addition, CM collected from VAT could induce the cancer cells to express higher levels of inflammation-related chemokines.

Since we previously found the involvement of leptin in inhibiting mitochondrial respiration and gene expression in HCT116 human colon cancer cells [12], we also addressed this issue in the present study. Accordingly, we measured higher leptin levels in CM prepared from VAT (Fig. 3E) and SAT (Fig. 3F) collected from mice fed HFD as compared to CM prepared from mice fed CD. As expected, leptin levels were higher in CM prepared from VAT or SAT of mice fed HFD as compared to mice fed CD. Additionally, leptin blood levels in mice fed HFD were also significantly higher than in those fed CD (Supplementary Fig. S3A).

### Co-culturing MC38 cells with VAT or SAT explants affects leptin secretion to the media, leptin expression in the cells, and gene expression related to inflammation and lipid metabolism

**Figure 4:**
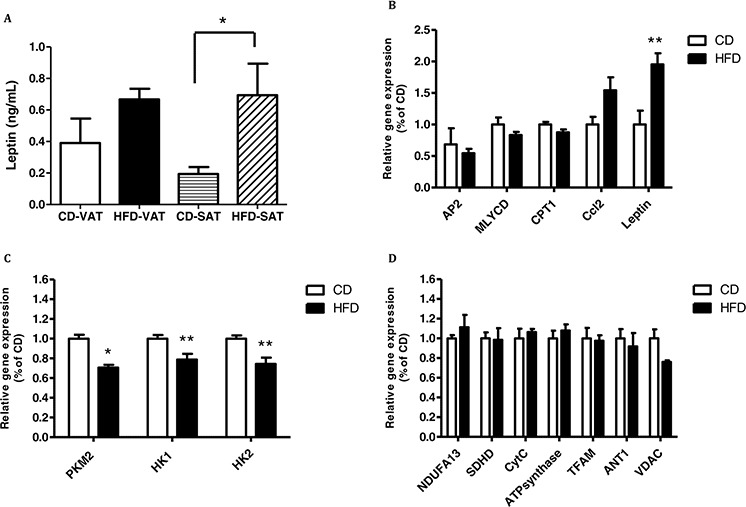
Co-culturing of MC38 cells with visceral adipose tissue (VAT) of high- fat diet (HFD)- fed mice increases the expression of leptin and decreases the expression of the glycolytic genes MC38 cells were treated with VAT or subcutaneous adipose tissue (SAT) explants for 24 hours. **A.** Leptin level was measured using an ELISA kit; *n* = 3–5 mice from each group, *P < 0.05 for HFD vs. control diet (CD) (Student's unpaired *t*-test). **B–D.** Gene expression levels were detected using quantitative real-time PCR in cells co-cultured with VAT. *P < 0.01, **P < 0.05 vs. CD mice; *n* = 4 (Student's unpaired *t*-test).

In exploring the cross talk between adipose tissue and cancer, we tested interactions between adipose tissue and cancer cell function. We used a co-culture system in which VAT or SAT explants were co-incubated with MC38 cells for 24 hours. At the end of the experiment, leptin levels in the media were measured, and cells were processed for mRNA analysis. As shown in Fig. 4A, MC38 cells co-cultured with SAT explants from mice fed HFD secreted significantly more leptin into the media than MC38 cells co-cultured with SAT explants of mice fed CD; this trend was also evident with VAT explants from mice fed HFD (Fig. 4A). In addition, we found significant upregulation of leptin transcription in MC38 cells exposed to VAT explants from mice fed HFD; this upregulatory effect was also observed for expression of the proinflammatory cytokine *Ccl2*. No effect was recorded on the expression levels of genes related to lipid metabolism in these MC38 cell samples (Fig. 4B).

**Figure 5:**
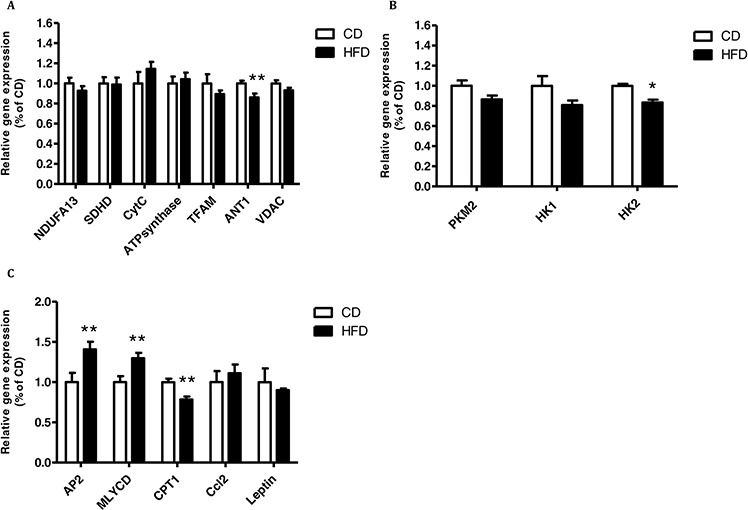
Co-culturing of MC38 cells with subcutaneous adipose tissue (SAT) changed the expression of mitochondrial and glycolytic genes and genes related to lipid metabolism MC38 cells were treated with SAT explants for 24 hours. **A–C.** Gene expression levels were detected using quantitative real-time PCR in cells co-cultured with SAT. *P < 0.01, **P < 0.05 vs. CD mice; *n* = 4 (Student's unpaired *t*-test).

Interestingly, co-culturing MC38 cells with VAT or SAT explants from mice fed HFD affected the expression of glycolytic genes more than that of mitochondrial genes (Fig. 4C,4D and Fig. 5A,5B). Co-culture of MC38 cells with VAT explants from mice fed HFD significantly reduced the expression levels of *PKM2*, *HK1* and *HK2* (Fig. 4C).

Co-culture of MC38 cells with SAT explants of mice fed HFD induced a significant decrease in the expression of expression of adenine nucleotide translocator (ANT1) and HK2 (Fig. 5A, 5B). In addition, there was a significant increase in the expression of malonyl-CoA decarboxylase (*MLYCD*) and *AP2* (encoding a carrier protein for fatty acid) and a significant decrease in the expression of carnitine palmitoyltransferase 1 (*CPT1*) (Fig. 5C). Trends were detected for *PKM2* but did not reach statistical significance (Fig. 5B).

### Cancer cells decrease mitochondrial complexes expression by regulating JNK/STAT3-signaling pathway

**Figure 6:**
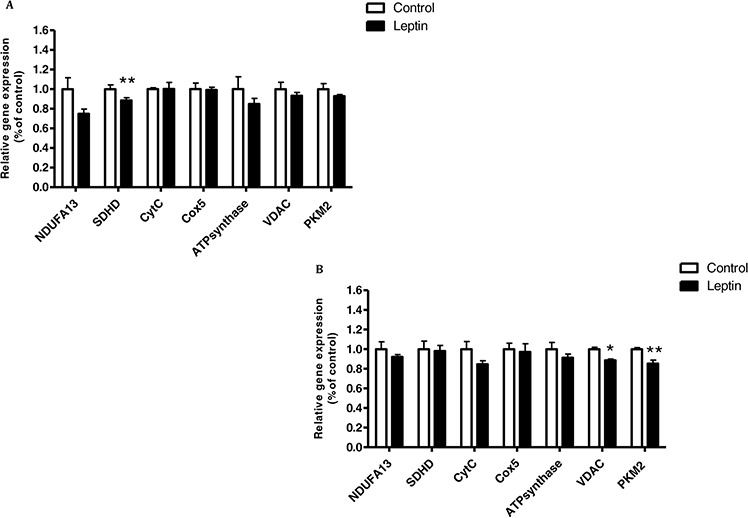
Leptin reduced mitochondrial function of MC38 and CT26 cells **A.** MC38 and **B.** CT26 cells were incubated with medium (control) or leptin (100 ng/mL) for 24 hours. Target transcript was detected using quantitative real time PCR. *P < 0.01, **P < 0.05 vs. control; *n* = 10 (Student's unpaired *t*-test). Three independent experiments were performed.

Since leptin is directly correlated with obesity [23] and cancer progression [14], and activates the JNK/STAT3-signaling pathway, we investigated whether leptin and the respective JNK/STAT3-signaling pathway [13, 24, 25] play some role in modulating the expression of mitochondrial or glycolytic genes. First, we determined the effect of leptin on the expression of mitochondrial and glycolytic genes in CT26 and MC38 cells. In the latter, leptin significantly decreased *SDHD* expression level. The same trend was shown for *NDUFA13* but did not reach statistical significance (*P* = 0.06) (Fig. 6A). In CT26 cells, leptin significantly downregulated the expression levels of the mitochondrial gene voltage-dependent anion channel (*VDAC*) and the glycolytic gene *PKM2* (Fig. 6B).

**Figure 7:**
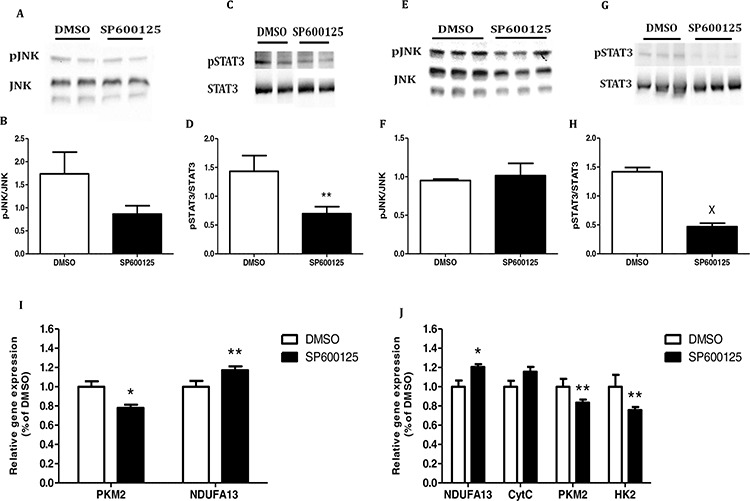
Inhibition of JNK/STAT3 signaling pathway re-establish the mitochondrial function **A–D.** MC38 and **E–H.** CT26 cells were starved for 24 hours and then incubated for 1 hour with DMSO (0.1% as control) or SP600125 (10 μM). Total cell lysates were analyzed by western blot and densitometry analysis of the western blot data was performed. **P < 0.05, ^X^*P* < 0.001 vs. control (Student's unpaired *t*-test). **I.** MC38 and **J.** CT26 cells were incubated in starvation medium with DMSO (0.1%) or SP600125 (10 μM) for 24 hours. All target transcripts were detected using quantitative real-time PCR; *n* = 7–8 for each treatment, **P < 0.05, *P < 0.01 vs. control (Student's unpaired *t*-test). DMSO (control) was set to 1. One of two independent experiments is presented.

We then treated the cells with the pJNK inhibitor SP600125. As expected, SP600125 significantly inhibited JNK and STAT3 phosphorylation in both cell lines (Fig. 7A–7H). Concomitantly, in MC38 cells, SP600125 significantly increased the expression of the mitochondrial gene *NDUFA13* (*P* < 0.05) and significantly decreased that of *PKM2* as compared to control cells (Fig. 7I).

CT26 cells treated with SP600125 showed a significant increase in *NDUFA13* expression compared to the control. The same trend was observed in the expression of Cytochrome complex *(CytC)*, but did not reach statistical significance. In addition, we demonstrated a significant decrease in *PKM2* and *HK2* expression as compared to the control (Fig. 7J).

### HFD feeding decreases mitochondrial activity in brown adipose tissue, liver and colonic tissues but does not affect white adipose tissue

Finally, to study the direct effect of obesity on mitochondrial function *in vivo*, and to correlate it to the above results, we tested several genes involved in metabolism in the colon, liver, brown adipose tissue (BAT) and white adipose tissue (WAT) of mice fed HFD and compared to those fed CD.

**Figure 8:**
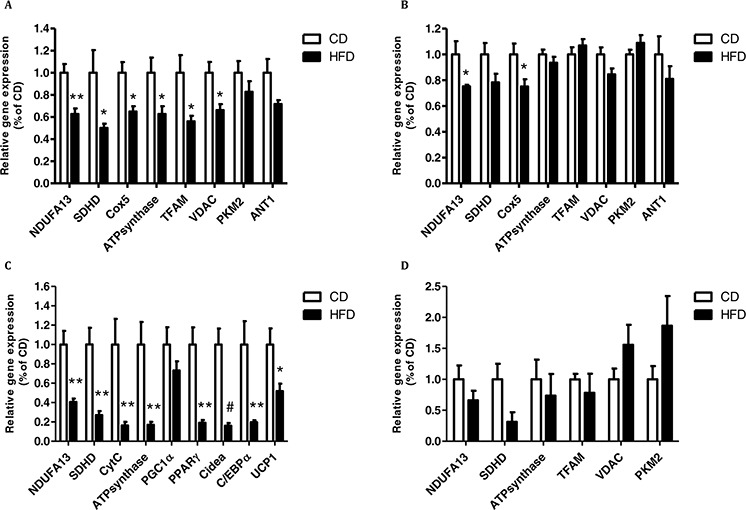
High- fat diet (HFD) promotes mitochondrial dysfunction in brown adipose tissue (BAT), and in liver and colon tissues Tissues from **A.** liver, **B.** colon, **C.** BAT and **D.** White adipose tissue (WAT) were collected from control diet (CD) and HFD mouse groups and gene expression level was detected using quantitative real-time PCR. A, B, *n* = 5 HFD mice, *n* = 4–5 CD mice. C, D, *n* = 8–9 CD and HFD mice. *P < 0.05, **P < 0.01, #*P* < 0.001 (Student's unpaired *t*-test).

Liver tissue collected from mice fed HFD showed substantially reduced expression of several mitochondrial marker genes and genes of mitochondrial complexes (Fig. 8A) as compared to mice fed CD. The colonic tissue produced the same trend in some of the genes but the effects were less pronounced than those observed in the liver (Fig. 8B). Surprisingly, in BAT we did see the same trend as in the liver in the expression of the mitochondrial complexes genes tested (Fig. 8C) nonetheless in WAT, no significant change in either mitochondrial or glycolytic genes as a result of HFD feeding was observed (Fig. 8D). Thus, when checking a core set of brown fat–specific genes, which are correlated to BAT thermogenic and adipogenesis ability [26, 27] such as the uncoupling protein 1 (*UCP1*), peroxisome proliferator-activated receptor gamma (PPARγ), cell death-inducing DFFA-like effector A *(Cidea)*, peroxisome proliferator-activated receptor-γ coactivator 1-α (*PGC1α)* and CCAAT-enhancer-binding protein α (*C/EBPα*) we showed that in all of the HFD mice tissues tested, there was a significant downregulation in the expression of these genes as compared to CD group except for *PGC1α* which its reduction did not reach significance (Fig. 8C).

## DISCUSSION

A direct relationship between obesity, adipose tissue-related factors and colon cancer has been hypothesized in recent years, but no definitive conclusions have been reached regarding the involved factors. This correlation is strongly supported by epidemiological evidence, which clearly indicates that the risk of developing colon cancer is enhanced by obesity, leading to a worse prognosis after diagnosis and to increased chances of mortality [8, 28, 29].

We approached this controversial issue using an *in-vivo* murine model of carcinogenesis (C57BL/6 mice) which was subjected to subcutaneous injection of syngeneic cell line MC38 into two groups of these mice: one fed CD and the other HFD. The difference between the body weight of the CD and HFD-fed mice was 4.8 g after subtracting the difference in tumor weight, which corresponds to 16.5% of body weight (data not shown). This percentage is responsible for the significant difference in epididymal fat mass. In the present study, we demonstrate that feeding mice with an HFD which promoted an increase in body weight and lead to insulin resistance can result in: (i) the development of larger tumors and concomitantly into faster tumor progression; (ii) mice tumor activities of HFD mice are associated with enhanced expression levels of PCNA and pJNK; proteins related to cell growth and proliferation [8, 19]. In line with these results, a direct correlation between mouse body weight and tumor weight was recorded. Similarly, Park *et al* [8] demonstrated that chronic consumption of HFD may stimulate colon cancer progression even in individuals who maintain healthy body weights, in their study they used BALB/c mice which are resistant to HFD induced obesity. Park *et al* [8] prepared their HFD with high amounts of animal fat. Their conclusions were that HFD containing animal fat should be avoided in order to prevent colon cancer. In our study we choose C57BL/6 mice which are immune-competent with MC38 colon cancer cells. The mice strain we elected is not resistant to HFD induced obesity; this feature gave us the opportunity to study in this model the general effect of overweight and obesity on tumor progression.

An interesting aspect of our findings was that even though the HFD we used contained palm oil and not animal fat, the effects obtained were significant towards weight gain as well as to colon cancer development. Palm oil is known to contain highly saturated vegetable fat. Thus, we assume that highly saturated fat from animal or vegetable source can equally promote obesity which can equally lead to colon cancer.

We conclude therefore that in the mouse model we used, there was a direct relationship between obesity and cancer, and therefore we could address the mechanisms involved in this *in-vivo* model.

Insulin resistance has been linked to many disease processes, among them diabetes mellitus, obesity, hypertension, dyslipidemia, and atherosclerotic cardiovascular disease [30]. Insulin resistance underpins the tight association between type 2 diabetes and obesity pandemics [31].

Previous studies have provided some evidence of an association between metabolic factors and increased risk of colorectal carcinogenesis [18, 23]. Sleigh *et al* [32] reported that obesity and insulin resistance are associated with mitochondrial dysfunction. We previously reported that colon cancer cells exposed to CM from obese subjects show a significant reduction in mitochondrial respiration rate and in the expression level of mitochondrial genes [12]. Therefore, one of the aims of the present study was to identify metabolic pathways that could correlate cancer-related obesity with mitochondrial dysfunction. To this end, we used an *ex-vivo* model in which we exposed murine colon cancer cells to adipose tissue preparations from mice fed CD or HFD. We have shown in our previous publication that by using an antagonist to the leptin receptor we could detect a significant protection against OCR reduction induced by CM collected from obese subjects in HCT116 human colon cancer cells [12]. To assess whether chemokines present in the CM preparations are responsible for the *in-vitro* effects in colon cancer cell lines, we measured leptin levels in the different CM preparations. Those from fat samples taken from mice fed HFD contained, as expected, more leptin than CM prepared from fat samples from mice fed CD. An interesting finding was that medium aliquots obtained after exposing the fat tissues to cancer cells contained higher levels of leptin than unexposed CM. A possible explanation for this interesting finding is that the cancer cells are triggered to secrete higher concentrations of leptin and other inflammatory cytokines. Support for this view was obtained when we measured the gene expression of the cytokines *leptin* and *Ccl2* in the cells exposed to VAT explants of mice fed HFD, as their expression was higher than in cells exposed to VAT explants of mice fed CD. Additional data to support these findings (data not shown) come from measurements conducted in our lab of the levels of leptin and Ccl2 in the media of HM-7 and HCT116 human colon cancer cells co-cultured with human adipose cells. The results of these experiments showed higher levels of these cytokines released to the culture media as compared to the levels in a basal media of the cancer cells or the adipose cells cultured alone, making these findings relevant to both, murine and human tissues.

Similar to our previous study [12], CT26 and MC38 colon cancer cells treated with CM prepared from VAT from obese mice demonstrated lower mitochondrial respiration, as reflected by the reduction in OCR and maximal OCR after adding the mitochondrial uncoupler FCCP. In contrast, CM prepared from SAT did not alter these metabolic parameters in either CT26 or MC38 colon cancer cells. Support to these results was obtained from measurements of the OCR/ECAR ratio which showed a significant decrease after adding VAT CM on MC38 and CT26 cells. However, CM prepared from VAT and SAT did not affect glycolysis since ECAR did not change.

Expression of relevant genes supported the results of mitochondrial respiration in CT26 cells. Exposure of these cells to CM obtained from VAT from mice fed HFD demonstrated significantly reduced expression of the mitochondrial genes, while there was no change in the glycolytic genes. However, in MC38 cells, we did not measure a significant change in the expression of either mitochondrial or glycolytic genes, even though a small but significant reduction in mitochondrial respiration was observed. We assume that the different responses of CT26 and MC38 colon cancer cells to CM from adipose tissue with respect to mitochondrial dysfunction are based on the extent of malignancy of the cancer cells. Adipose tissue secretions induce metabolic alterations such as mitochondrial dysfunction that ultimately induce the cell to become more malignant. We assume that if the cancer cells are in an advanced stage of malignancy, treatment with CM will not further alter their already non-functional metabolism. Supporting this view, Melero *et al* [33] previously demonstrated that injecting mice with bone marrow- derived dendritic cells genetically engineered to produce high levels of functional IL-12, induces complete regression in 84% of CT26-derived subcutaneous tumor nodules as compared to MC38 cell-derived tumors, where only 50% of the tumor nodules were affected. We surmise that a malignant cell line such as MC38 has already reached its maximal metastasis and proliferation levels, which can be associated to aglycemia and nutrient shortage. This condition may lead to stimulating glutaminolysis, which can partially reestablish OXPHOS as an adaptation response [34]. In addition, *Ccl2* and *IKK-_e_* genes were upregulated in MC38 cells when adding HFD CM from VAT, and there was no change in their levels in CT26 cells. This result further supports the fact that MC38 cells are more malignant than CT26 cells.

The unchanged trend neither in the expression of the glycolitic genes nor in the ECAR levels after exposing to CM can be explained by the fact that many tumors produce most of their ATP by mitochondrial respiration [34, 35]. Some reports support the fact that cancer cells use mitochondrial respiration and ATP production to stand for their energy demands, instead of using glycolisis which is much less efficient than OXPHOS for producing ATP. Thus, not all findings support the fact that glycolitic changes happened along with tumorigenesis [34–36].

In the co-culture experiments, MC38 cells exposed directly to adipose tissue explants harvested from VAT and SAT of mice fed HFD showed different effects. All of the glycolytic genes were downregulated, whereas for the mitochondrial genes, only *VDAC* and *ANT1* were downregulated. We assume that the reduction in expression of the glycolytic genes is due to the high level of fatty acids released to the MC38 culture media, especially from the explants of mice fed HFD, inducing increased fatty acid oxidation. In the case of SAT this assumption is supported by the higher measured expression of *AP2* and *MLYCD*. *MLYCD* promotes oxidation in the peroxisome, this process can increase the intramitochondrial acetyl-CoA/CoA and NADH/NAD ratios and subsequently inactivate pyruvate dehydrogenase. As a result, the citrate concentration increases, leading to inhibition of phosphofructokinase and subsequent accumulation of glucose-6-phosphate. Higher concentrations of the latter inhibit HK2, resulting in decreased glucose uptake [37, 38]. We think that the oxidation process only takes place in the peroxisome, since the expression of *CPT1*—the carrier protein of fatty acid into the mitochondria—was downregulated and this can be a direct result of acetyl-CoA/CoA increase. Therefore, as a result of HFD, the mitochondria losses its ability to oxidize fatty acid so most of the secreted fatty acid do not oxidize as it should in the mitochondria, as a result, we did not see a higher OCR as should be expected when fatty acid oxidize, alternately we did see a lower OCR and lower expression of mitochondrial and glycolitic genes when co culturing the cells with HFD tissue fat explants.

To shed light of the effects exerted by VAT we measured in MC38 cells the effect of CM prepared from VAT explants of mice fed CD or CM prepared from VAT explants of mice fed HFD on OCR and ECAR activities compared to the effects of control media (data not shown). We found in these experiments that there was a reduction in the OCR levels either after adding CM prepared from VAT explants of mice fed CD or CM prepared from VAT explants of mice fed HFD as compared to the control media. However, the reduction was much more significant when the MC38 cells were treated with CM prepared from VAT explants of mice fed HFD. This result may indicate that CM, especially if it is prepared from VAT explants of mice fed HFD contain higher levels of soluble factors that are secreted to the media by the fat explants which can affect both mitochondrial and glycolitic activities.

In the present study, we demonstrated that leptin downregulated *SDHD* and *NDUFA13* expression in MC38 cells and *VDAC* expression in CT26 cells, similar to our previous study [12]. We then analyzed whether the signaling pathway associated with leptin plays a role in the obesity-related mitochondrial dysfunction observed in colon cancer. We hypothesized that the JNK/STAT3-signaling pathway which is one of the main mechanisms activated by leptin and associated with excess proliferation and inhibition of apoptosis [39, 40] plays a role in the obesity-related mitochondrial dysfunction observed in colon cancer. In MC38 cells treated with the pJNK inhibitor SP600125, the expression level of the glycolytic gene *PKM2* was significantly reduced and concomitantly the level of the mitochondrial gene *NDUFA13* increased. In CT26 cells, we obtained a similar trend, i.e., expression of *PKM2* and *HK2* was significantly reduced whereas that of *NDUFA13* increased. We conclude that the JNK/STAT3-signaling pathway induced by leptin is indeed involved in the mitochondrial dysfunction related to obesity in colon cancer cells.

An additional salient finding of our study is the significant decrease in the expression of mitochondrial genes in different organs such as liver, colon and BAT obtained from mice fed HFD and developed insulin resistance as compared to mice fed CD. These results are in line with the results of our *in vitro* and *ex vivo* experiments and are also supported by recent observations by Yuzefovych *et al* [2] who showed that a HFD downregulates the expression of mitochondrial genes in mouse liver and muscle. Nonetheless, and as expected we did not see any change in gene expression in WAT. During excessive nutritional overload, adipocytes in WAT and cells of the skeletal muscle contain low levels of ATP and concomitantly accumulate NADH [41, 42]. This metabolic pathway shift towards enhanced lipid storage, reduced mitochondrial biogenesis and enhanced glycolytic ATP synthesis results in additional lipid accumulation and the potential for subsequent progression towards insulin resistance. BAT in contrast to WAT; participate in energy dissipation through heat rather than via energy storage. We show that a HFD promoted a significant reduction in the expression levels of four mitochondrial complexes genes. Our results are in line to those of Mercer *et al* [43], where they found that the total cytochrome oxidase activity in BAT (an index of mitochondrial mass) was lower in the ob/ob mice fed a low fat diet or a beef tallow diet than in lean littermates fed the same diet. Moreover, the reduction in the expression of several BAT genes as *UCP1*, *PPARγ*, *C/EBPα* and *Cidea* computing the final phase during early postnatal life, which characterized by the loss of *UCP1* accompanied by a decline in genes primarily associated with BAT. This results in increasing characteristics of white adipose tissue [27]. Thus, obesity can promote mitochondrial dysfunction in BAT causing a reduction in BATs ability to perform a complete thermogenesis. Finally, these results suggest that the adipose tissue may affect metabolic parameters of visceral–proximal tissues, such as the colon and the liver, in a way that might lead to the onset of tumors in these tissues.

We conclude from this study that (i) obesity induces mitochondrial dysfunction, promoting cancer in several organs adjacent to the visceral fat, such as the colon, and (ii) inhibition of the JNK/STAT3-signaling pathway in colon cancer cells seems to be critical for reestablishing mitochondrial function and overcoming the glycolytic phenotype. We assume that this signaling pathway may be partly responsible for the relation between obesity–leptin-induced mitochondrial dysfunction and colon cancer.

## MATERIALS AND METHODS

### Materials

All chemicals and biochemicals were from Sigma Chemical Co. (St. Louis, MO, USA), unless otherwise specified.

### Cell culture

CT26 and MC38 are colon adenocarcinoma cell lines of BALB/c and C57BL/6 origin, respectively [44]. MC38 cells were maintained in Dulbecco's Modified Eagle Medium (DMEM) containing 100 mL/L fetal calf serum (FCS) with 100,000 U/L penicillin and 100 mg/L streptomycin, 1 mM sodium pyruvate, 2 mM L-glutamine and 1 mM non-essential amino acid solution. CT26 cells were maintained in RPMI 1640 containing 100 mL/L FCS with 100,000 U/L penicillin and 100 mg/L streptomycin. Cells were kept at 37°C with 5% CO_2_.

Cells were seeded into 12-well plates (2 × 10^5^ cell/well) for 24 hours before incubation with CM for an additional 24 hours. Then, RNA was extracted as described below.

In studies involving the pJNK inhibitor SP610025, cells were cultured to confluence in 6-well plates, and treated with SP610025 (10 μM) or DMSO (0.1% v/v) as a control in starvation media for another 24 hours for RNA extraction. For protein extraction, cells were starved for 24 hours and treated for 1 hour with 10 μM SP610025 or 0.1% DMSO.

### Animals, experimental diets and injection of MC38 colon cancer cells

Experiment 1: 7 to 8 weeks old C57BL/6 male mice, weighing 20–26 g were purchased from Harlan (Jerusalem, Israel). A total of 40 mice were used, 10 mice in each group. Animals were housed in the animal facility, kept under standard conditions at a constant temperature of 24°C, and provided *ad libitum* access to food and water. After 1 week of acclimatization, mice were randomly divided into two dietary groups, a control group which was fed a control diet (CD) (10% kcal from fat) or a high-fat diet (HFD) group which was fed HFD (60% kcal from fat) for a total experimental period of 18 weeks. The dietary composition is provided in Supplementary Table S2.

Fourteen weeks after administration of the diets, MC38 cells (10^5^ cells suspended in 0.1 mL DMEM) were subcutaneously injected into the right rear flank of mice fed HFD or CD (9–10 mice from each group) and the dietary intervention was continued for an additional 4 weeks. The width (a) and length (b) of the growing tumors were measured three times a week, and the corresponding volume (V) was calculated using the equation V = (a^2^ x b)/2. After 18 weeks, mice were sacrificed, tumor tissues were excised and weighed, and tumor aliquots were transferred into 4% paraformaldehyde solution for histology or immunohistology or into lysis buffer for western blot analysis.

Experiment 2: This experiment was conducted in order to obtain CM and for co-culture experiments. To this end we used 5- to 6-week-old male C57BL/6 mice (*n* = 20) weighing 16–20 g. After 1 week of acclimatization, mice were randomly divided into CD and HFD groups (Supplementary Table S2). Mice were fed for 16 weeks the experimental diets (CD, HFD), and body weight and food intake were registered three times a week during the experiment. Animal care and experimental procedures in both experiments were in accordance with the guidelines of the animal ethics committee of the Hebrew University of Jerusalem.

### Plasma glucose concentration tolerance test

An intraperitoneal glucose tolerance test was performed on overnight-fasted animals after 6 and 12 weeks to confirm their glucose-tolerant status. Plasma glucose concentrations were measured in blood samples obtained from the tail using test reagent strips (Freestyle, Abbott, Witney, UK) prior to, and 30, 60, 90 and 120 minutes after intraperitoneal injection of 2 g/kg body weight D-glucose (200 mg/mL) for the glucose tolerance test.

### CM preparation

Visceral adipose tissue (VAT) and subcutaneous adipose tissue (SAT) from C57BL/6 mice fed for 16 weeks with CD (*n* = 5) or HFD (*n* = 5) was collected from mice of Experiment 2. Adipose tissue explants (1–3 mm^3^, 100 mg/mL medium) which were collected were obtained and incubated at 37°C in DMEM with 10% (v/v) FCS and 2 mM L-glutamine. They were allowed to settle overnight, the medium was replaced, and explants were further incubated for 24 hours in the same medium without FCS. The explants were removed with tweezers, and CM were transferred and quickly frozen (10 seconds) in liquid nitrogen for storage at −80°C.

### Co-culture experiment

VAT or SAT explants (1–3 mm^3^) (collected from mice of Experiment 2) were incubated under sterile conditions for at least 30 minutes in saline and then placed above MC38 mouse colon cancer cells growing in a 12-well plate (2 × 10^5^ cell/well) with DMEM containing 0.1% (v/v) FCS. After 24 hours, the medium was tested for leptin by ELISA and the MC38 cells were collected for RNA extraction.

### Cell respiration measurements

Cellular oxygen consumption rate (OCR) and extracellular acidification rate (ECAR) were measured using the XF24 Analyzer (Seahorse Bioscience, Massachusetts, USA) as described previously [45]. For maximal OCR, 0.4 μM carbonyl cyanide-4-trifluoromethoxy-phenylhydrazone (FCCP) was used. Optimal FCCP concentration was determined in preliminary experiments. OCR/ECAR ratio was also presented.

### RNA extraction and real-time PCR

RNA was isolated using Tri Reagent solution (Sigma-Aldrich, St. Louis, MO, USA). Reverse transcription was performed using a High-Capacity cDNA Kit (Applied Biosystems, Foster City, CA, USA) with random primers on a Veriti^®^ 96-well Thermal Cycler (Applied Biosystems). Real-time PCR was performed using SYBR^®^ Green (Applied Biosystems) in an ABI PRISM^®^ 7300.

Primers are listed in Supplementary Table S3. All results were normalized to the expression of the *β-actin* gene, except for genes expression in white adipose tissue (WAT) and brown adipose tissue (BAT) which were normalized to the *Cox5* gene.

### Western blotting

Tissues or cells were lysed in RIPA buffer (50 mM Tris–HCl pH 7.4–7.6, 150 mM NaCl, 4 mM EDTA, 1% v/v Triton X-100, 0.1% v/v SDS, 1% v/v sodium deoxycholate, 1 mM phenylmethylsulfonyl fluoride, 5 mM NaF, 2 mM sodium orthovanadate, and 1% v/v protease inhibitor cocktail pH 7.4) and centrifuged at 23,000*g* for 15 minutes. Protein concentration was determined in the supernatant by bicinchoninic acid-based protein assay (BCA) (Pierce, Rockford, IL, USA). Samples (50 μg protein) were subjected to SDS-PAGE, transferred to nitrocellulose membranes (Whatman, Schleicher & Schuell, Dassel, Germany) and blocked in 5% (w/v) dry nonfat milk (Difco, Sparks, MD, USA) or 5% (w/v) bovine serum albumin in PBS containing 0.05% (v/v) Tween-20, as described previously [19]. Membranes were incubated with primary mouse polyclonal antibodies: anti-pJNK (Santa Cruz Biotechnology, Dallas, TX, USA), anti-JNK (Santa Cruz), or rabbit polyclonal antibodies: anti-pSTAT3 (Cell Signaling Technology, Danvers, MA, USA), anti-STAT3 (Santa Cruz), anti-β-actin (Sigma-Aldrich). Secondary antibodies were obtained from Jackson ImmunoResearch (West Grove, PA, USA). Proteins were visualized using an enhanced chemiluminescence kit (Santa Cruz).

### Immunohistochemistry

Tissue (5-μm thick sections) was processed, incubated with the primary antibody anti-proliferating cell nuclear antigen (PCNA) (Santa Cruz Biotechnology, Dallas, TX, USA) and examined with an inverted light microscope (Nikon Eclipse E400) at X40 magnification as described previously [31].

### ELISA

Insulin and leptin levels were determined using ELISA kits (Leptin ELISA Kit MOB00, R&D Systems, Minneapolis, USA; Insulin ELISA 10–1113-01, Mercodia, Uppsala, Sweden) according to the manufacturers' instructions.

### Cell viability

Cell viability was measured using Neutral red lysosomal uptake assay as previously described [46].

### Data analysis

Depending on the experimental settings, statistical analyses were performed by one- or two-way repeated-measure ANOVA with Tukey-Kramer or Bonferroni's *Post hoc* tests, or Student's unpaired *t*-test as specified in the figure legends. Statistical analyses were done using GraphPad Prism software and the *p* values indicated. Results are presented as mean ± SEM. All figures show representative results of at least two independent experiments.

## SUPPLEMENTARY MATERIAL FIGURES AND TABLES



## References

[1] Khan S, Shukla S, Sinha S, Meeran SM (2013). Role of adipokines and cytokines in obesity-associated breast cancer: therapeutic targets. Cytokine Growth Factor Rev.

[2] Yuzefovych LV, Musiyenko SI, Wilson GL, Rachek LI (2013). Mitochondrial DNA damage and dysfunction, and oxidative stress are associated with endoplasmic reticulum stress, protein degradation and apoptosis in high fat diet-induced insulin resistance mice. PLoS One.

[3] Liu HY, Yehuda-Shnaidman E, Hong T, Han J, Pi J, Liu Z, Cao W (2009). Prolonged exposure to insulin suppresses mitochondrial production in primary hepatocytes. J Biol Chem.

[4] Santidrian AF, Matsuno-Yagi A, Ritland M, Seo BB, LeBoeuf SE, Gay LJ, Yagi T, Felding-Habermann B (2013). Mitochondrial complex I activity and NAD+/NADH balance regulate breast cancer progression. J Clin Invest.

[5] Kamp DW, Shacter E, Weitzman SA (2011). Chronic inflammation and cancer: the role of the mitochondria. Oncology (Williston Park).

[6] Aleman JO, Eusebi LH, Ricciardiello L, Patidar K, Sanyal AJ, Holt PR (2014). Mechanisms of obesity-induced gastrointestinal neoplasia. Gastroenterology.

[7] Sung MK, Bae YJ (2010). Linking obesity to colorectal cancer: application of nutrigenomics. Biotechnol J.

[8] Park H, Kim M, Kwon GT, Lim do Y, Yu R, Sung MK, Lee KW, Daily, JW, Park JH (2012). A high-fat diet increases angiogenesis, solid tumor growth, and lung metastasis of CT26 colon cancer cells in obesity-resistant BALB/c mice. Mol Carcinog.

[9] Dirat B, Bochet L, Dabek M, Daviaud D, Dauvillier S, Majed B, Wang YY, Meulle A, Salles B, Le Gonidec S, Garrido I, Escourrou G, Valet P, Muller C (2011). Cancer-associated adipocytes exhibit an activated phenotype and contribute to breast cancer invasion. Cancer Res.

[10] Nieman KM, Kenny HA, Penicka CV, Ladanyi A, Buell-Gutbrod R, Zillhardt MR, Romero IL, Carey MS, Mills GB, Hotamisligil GS, Yamada SD, Peter ME, Gwin K, Lengyel E (2011). Adipocytes promote ovarian cancer metastasis and provide energy for rapid tumor growth. Nat Med.

[11] Tebbe C, Chhina J, Dar SA, Sarigiannis K, Giri S, Munkarah AR, Rattan R (2014). Metformin limits the adipocyte tumor-promoting effect on ovarian cancer. Oncotarget.

[12] Yehuda-Shnaidman E, Nimri L, Tarnovscki T, Kirshtein B, Rudich A, Schwartz B (2013). Secreted human adipose leptin decreases mitochondrial respiration in HCT116 colon cancer cells. PLoS One.

[13] Nowakowska-Zajdel E, Mazurek U, Stachowicz M, Niedworok E, Fatyga E, Muc-Wierzgon M (2011). Cellular signal transduction pathways by leptin in colorectal cancer tissue: preliminary results. ISRN Endocrinol.

[14] Jaffe T, Schwartz B (2008). Leptin promotes motility and invasiveness in human colon cancer cells by activating multiple signal-transduction pathways. Int J Cancer.

[15] Endo H, Hosono K, Uchiyama T, Sakai E, Sugiyama M, Takahashi H, Nakajima N, Wada K, Takeda K, Nakagama H, Nakajima A (2011). Leptin acts as a growth factor for colorectal tumours at stages subsequent to tumour initiation in murine colon carcinogenesis. Gut.

[16] Montague CT, Prins JB, Sanders L, Zhang J, Sewter CP, Digby J, Byrne CD, O'Rahilly S (1998). Depot-related gene expression in human subcutaneous and omental adipocytes. Diabetes.

[17] Klimcakova E, Roussel B, Marquez-Quinones A, Kovacova Z, Kovacikova M, Combes M, Siklova-Vitkova M, Hejnova J, Sramkova P, Bouloumie A, Viguerie N, Stich V, Langin D (2011). Worsening of obesity and metabolic status yields similar molecular adaptations in human subcutaneous and visceral adipose tissue: decreased metabolism and increased immune response. J Clin Endocrinol Metab.

[18] Yehuda-Shnaidman E, Schwartz B (2012). Mechanisms linking obesity, inflammation and altered metabolism to colon carcinogenesis. Obes Rev.

[19] Nimri L, Barak H, Graeve L, Schwartz B (2013). Restoration of caveolin-1 expression suppresses growth, membrane-type-4 metalloproteinase expression and metastasis-associated activities in colon cancer cells. Mol Carcinog.

[20] Cross RS, Malaterre J, Davenport AJ, Carpinteri S, Anderson RL, Darcy PK, Ramsay RG (2015). Therapeutic DNA vaccination against colorectal cancer by targeting the MYB oncoprotein. Clinical & translational immunology.

[21] Chandra D, Singh KK (2011). Genetic insights into OXPHOS defect and its role in cancer. Biochim Biophys Acta.

[22] Cook CC, Higuchi M (2012). The awakening of an advanced malignant cancer: an insult to the mitochondrial genome. Biochim Biophys Acta.

[23] Schwartz B, Yehuda-Shnaidman E (2014). Putative role of adipose tissue in growth and metabolism of colon cancer cells. Front Oncol.

[24] Nalabolu MR, Palasamudram K, Jamil K (2014). Adiponectin and leptin molecular actions and clinical significance in breast cancer. Int J Hematol Oncol Stem Cell Res.

[25] Paz-Filho G, Mastronardi C, Franco CB, Wang KB, Wong ML, Licinio J (2012). Leptin: molecular mechanisms, systemic pro-inflammatory effects, and clinical implications. Arq Bras Endocrinol Metabol.

[26] Harms M, Seale P (2013). Brown and beige fat: development, function and therapeutic potential. Nat Med.

[27] Symonds ME (2013). Brown adipose tissue growth and development. Scientifica.

[28] Bardou M, Barkun AN, Martel M (2013). Obesity and colorectal cancer. Gut.

[29] Comstock SS, Hortos K, Kovan B, McCaskey S, Pathak DR, Fenton JI (2014). Adipokines and obesity are associated with colorectal polyps in adult males: a cross-sectional study. PLoS One.

[30] Samuel VT, Shulman GI (2012). Mechanisms for insulin resistance: common threads and missing links. Cell.

[31] Algamas-Dimantov A, Yehuda-Shnaidman E, Hertz R, Peri I, Bar-Tana J, Schwartz B (2014). Prevention of diabetes-promoted colorectal cancer by (n-3) polyunsaturated fatty acids and (n-3) PUFA mimetic. Oncotarget.

[32] Sleigh A, Raymond-Barker P, Thackray K, Porter D, Hatunic M, Vottero A, Burren C, Mitchell C, McIntyre M, Brage S, Carpenter TA, Murgatroyd PR, Brindle KM, Kemp GJ, O'Rahilly S, Semple RK (2011). Mitochondrial dysfunction in patients with primary congenital insulin resistance. J Clin Invest.

[33] Melero I, Duarte M, Ruiz J, Sangro B, Galofre J, Mazzolini G, Bustos M, Qian C, Prieto J (1999). Intratumoral injection of bone-marrow derived dendritic cells engineered to produce interleukin-12 induces complete regression of established murine transplantable colon adenocarcinomas. Gene Ther.

[34] Smolkova K, Plecita-Hlavata L, Bellance N, Benard G, Rossignol R, Jezek P (2011). Waves of gene regulation suppress and then restore oxidative phosphorylation in cancer cells. Int J Biochem Cell Biol.

[35] Zu XL, Guppy M (2004). Cancer metabolism: facts, fantasy, and fiction. Biochemical and biophysical research communications.

[36] Bluemlein K, Gruning NM, Feichtinger RG, Lehrach H, Kofler B, Ralser M (2011). No evidence for a shift in pyruvate kinase PKM1 to PKM expression during tumorigenesis. Oncotarget.

[37] Roden M (2004). How free fatty acids inhibit glucose utilization in human skeletal muscle. News Physiol Sci.

[38] Roden M, Price TB, Perseghin G, Petersen KF, Rothman DL, Cline GW, Shulman GI (1996). Mechanism of free fatty acid-induced insulin resistance in humans. J Clin Invest.

[39] Ogunwobi OO, Beales IL (2007). The anti-apoptotic and growth stimulatory actions of leptin in human colon cancer cells involves activation of JNK mitogen activated protein kinase, JAK2 and PI3 kinase/Akt. Int J Colorectal Dis.

[40] Saxena NK, Sharma D (2013). Multifaceted leptin network: the molecular connection between obesity and breast cancer. J Mammary Gland Biol Neoplasia.

[41] De Pauw A, Tejerina S, Raes M, Keijer J, Arnould T (2009). Mitochondrial (dys)function in adipocyte (de)differentiation and systemic metabolic alterations. The American journal of pathology.

[42] Muoio DM, Newgard CB (2006). Obesity-related derangements in metabolic regulation. Annual review of biochemistry.

[43] Mercer SW, Trayhurn P (1987). Effect of high fat diets on energy balance and thermogenesis in brown adipose tissue of lean and genetically obese ob/ob mice. The Journal of nutrition.

[44] Tirapu I, Arina A, Mazzolini G, Duarte M, Alfaro C, Feijoo E, Qian C, Chen L, Prieto J, Melero I (2004). Improving efficacy of interleukin-12-transfected dendritic cells injected into murine colon cancer with anti-CD137 monoclonal antibodies and alloantigens. Int J Cancer.

[45] Collins S, Yehuda-Shnaidman E, Wang H (2010). Positive and negative control of Ucp gene transcription and the role of beta-adrenergic signaling networks. Int J Obes (Lond).

[46] Repetto G, del Peso A, Zurita JL (2008). Neutral red uptake assay for the estimation of cell viability/cytotoxicity. Nat Protoc.

