# Supplementary material for: Mechanisms linking obesity to altered metabolism in mice colon carcinogenesis
Source: Oncotarget. 2015 Oct 12;6(35):38195–209. doi: 10.18632/oncotarget.5561 (PMC4741993; doi:10.18632/oncotarget.5561)
Supplement: Supplementary file 1 [file oncotarget-06-38195-s001.pdf]

SUPPLEMENTARY FIGURES AND TABLES

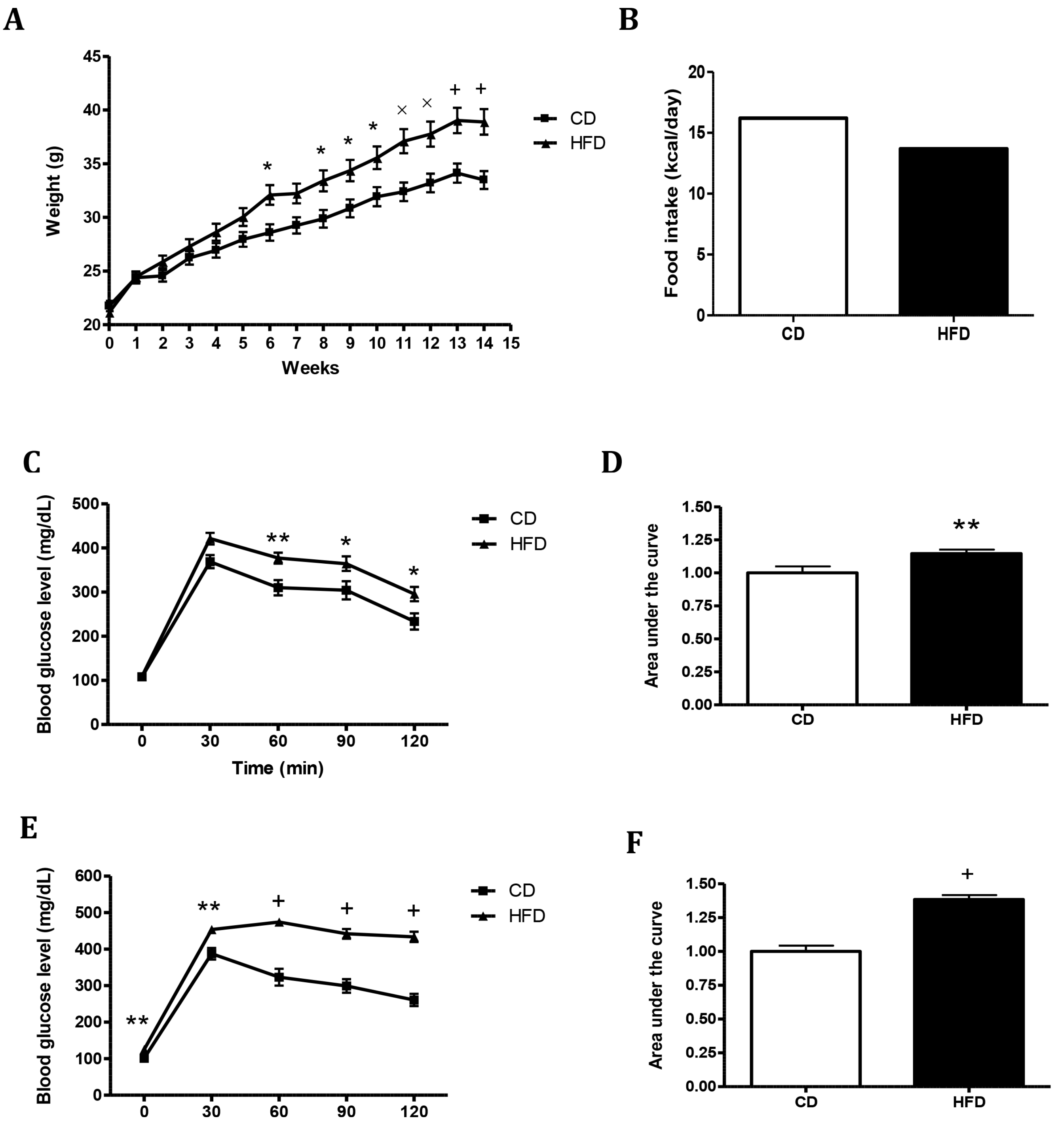

Supplementary Figure S1: High-fat diet (HFD) promotes weight gain and insulin resistance. Male 7-8 weeks old C57BL/6 mice were fed a control diet (CD) or a HFD for 18 weeks. After 14 weeks, an intraperitoneal glucose tolerance test was performed and 9–10 mice from each diet group were taken randomly and injected with 10^5 MC38 cells. The mice were fed the same diets continuously. All mice were sacrificed 31 days after the cells injection and tumors were isolated. **A.** Body weight were recorded for 14 weeks (until cells injection); $n = 19$ HFD mice, $n = 20$ CD mice, $^*P < 0.05$, $^xP < 0.01$, $^+P < 0.001$ vs. CD group (two-way ANOVA – Bonferroni post-test). **B.** Food intake was determined per mouse every 3 days (kcal/day). IPGTT and area under the curve were performed 6 **C, D.** and 12 **E, F.** weeks after starting the diets. C,E, $n = 19$ HFD, $n = 20$ CD mice, $^*P < 0.05$, $^{**}P < 0.01$, $^+P < 0.0001$ (two-way ANOVA – Bonferroni post-test). D, F. $n = 19$ HFD, $n = 20$ CD mice, $^{**}P < 0.01$, $^+P < 0.0001$ (Student's unpaired *t*-test).

Supplementary Table S1: Physiological parameters of different mice groups

	n	CD	HFD	*P* value
Weight (g)	9	30.31 ± 1.67	35.8 ± 1.60	<0.05
Fat mass weight (g)	9	1.06 ± 0.19	2.49 ± 0.27	<0.001
Leptin (ng/mL)	9	3.86 ± 0.57	13.10 ± 2.66	<0.01

Mice were treated as described in the Materials and Methods section (Experiment 1). Mice were sacrificed and their epididymal fat pads were weighed. Blood samples were collected, sera were prepared and leptin levels were measured using an ELISA kit; n = 9 mice from each group (Student's unpaired ***t***-test).

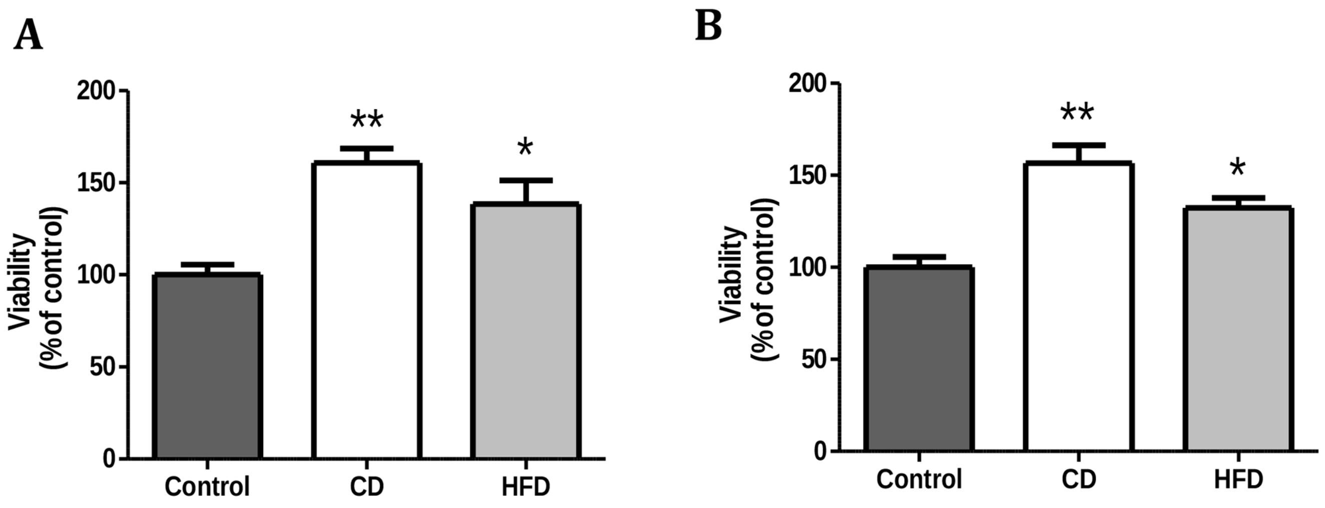

Supplementary Figure S2: Conditioned media (CM) supports cell viability. MC38 cells (5 × 10^4)were treated for 24 hours with visceral adipose tissue (VAT) CM **A.** or subcutaneous adipose tissue (SAT) CM **B.** obtained from mice fed a high- fat diet (HFD) or control diet (CD) (Experiment 2 in Materials and Methods). DMEM without fetal calf serum was used as a control. Cell viability was measured by Neutral red assay as described in Materials and Methods. *$P < 0.05$ and **$P < 0.01$ vs. control, $n = 5$ (one-way ANOVA – Tukey–Kramer).

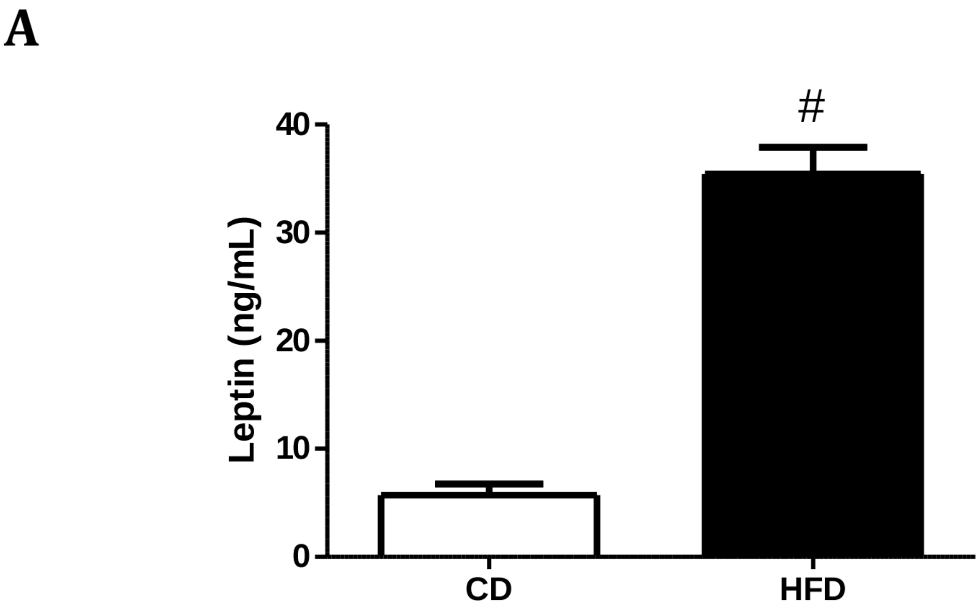

Supplementary Figure S3: Blood leptin levels in mice groups. Blood samples were collected from mice fed a control diet (CD) or high- fat diet (HFD) (Experiment 2 in Materials and Methods) and sera were prepared. Leptin levels (ng/ml) were measured using an ELISA kit; n = 10–11 mice from each group, $^{\#}P < 0.001$ (Student's unpaired t-test).

Supplementary Table S2: Diets given to the two mouse groups in the two experiments

Ingredient	CD (g/kg diet)	HFD (g/kg diet)
Casein	200	200
Cornstarch	506.2	0
Sucrose	68.8	68.8
Dextrimaltose	125	125
Cellulose	50	50
Soybean oil	25	25
Palm oil	20	245
Vitamin mix	10	10
Mineral mix	40	40
DL-Methionine	3	3
Choline bitartrate	2	2
BHT	0.014	0.014

The amounts of the ingredients in each diet are the amounts used to prepare 1 kg of diet. Most of the ingredients were purchased from Sigma-Aldrich or ENCO Ltd.

Supplementary Table S3: Mouse primers used in the real-time PCR experiments

Gene	Forward primer	Reverse primer
NDUFA13	5′-CGACTACAAGCGGAACCTG-3′	5′-TCCAGTAGCCAAAGATCAAGG-3′
SDHD	5′-CCTGCTCTGTGGTGGACTACT-3′	5′-CCCATGAACGTAGTCGGTAAC-3′
CytC	5′-GCTACCCATGGTCTCATCGT-3′	5′-CATCATCATTAGGGCCATCC-3′
Cox5	5′-GCTGCATCTGTGAAGAGGACAAC-3′	5′-CAGCTTGTAATGGGTTCCACAGT-3′
ATPsynthase	5′-ACCTATCCCAGCCTCGTCTC-3′	5′-AGGACTTGCCCACTTCTCTTT-3′
TFAM	5′-GTCCATAGGCACCGTATTGC-3′	5′-CCCATGCTGGAAAAACACTT-3′
VDAC	5′-GAGTATGGGCTGACGTTTACAG-3′	5′-GAGCTTCAGTCCACGAGCAAG-3′
ANT1	5′-GGCTCCTTCATCTTTTGCAATCT-3′	5′-GTAGGATGATGATGCAGTCTGG-3′
PKM2	5′-GTGGCTCGGCTGAATTTCTCT-3′	5′-CACCGCAACAGGACGGTAG-3′
HK2	5′-GATGGACCTCTTGGCATTTC-3′	5′-GGGACAAAAGTCCCAGTCTCT-3′
HK1	5′-GTGGACGGGACGCTCTAC-3′	5′-TTCACTGTTTGGTGCATGATT-3′
Leptin	5′-CCTCATCAAGACCATTGTCACC-3′	5′-TCTCCAGGTCATTGGCTATCTG-3′
MLYCD	5′-GTTCTCCTCCGGCTTCCT-3′	5′-GTTTTTCACAGGGTGCACAG-3′
AP2	5′-GATGCCTTTGTGGGAACCT-3′	5′-CTGTCGTCTGCGGTGATTT-3′
CPT1	5′-TGCCTTTACATCGTCTCCAA-3′	5′-GGCTCCAGGGTTCAGAAAGT-3′
Ccl2	5′-TTAAAAACCTGGATCGGAACCAA-3′	5′-GCATTAGCTTCAGATTTACGGGT-3′
IKK-$_{\varepsilon}$	5′-ACAAGGCCCGAAACAAGAAAT-3′	5′-ACTGCGAATAGCTTCACGATG-3′
β-actin	5′-TCGTTACCACAGGCATTGTGAT-3′	5′-TGCTCGAAGTCTAGAGCAAC-3′
